# Harnessing deep learning and statistical shape modelling for three‐dimensional evaluation of joint bony morphology

**DOI:** 10.1002/jeo2.70070

**Published:** 2024-10-26

**Authors:** Jacob F. Oeding, Allen A. Champagne, Eoghan T. Hurley, Kristian Samuelsson

**Affiliations:** ^1^ Department of Orthopaedics, Institute of Clinical Sciences, The Sahlgrenska Academy University of Gothenburg Gothenburg Sweden; ^2^ Department of Orthopaedic Surgery Duke University Medical Center Durham North Carolina USA

**Abbreviations**
3D: three‐dimensional
ACL: anterior cruciate ligament
AI: artificial intelligence
CT: computed tomography
DL: deep learning
MRI: magnetic resonance imaging
PCA: principal component analysis
SSM: statistical shape modelling

Medical research has continued to evolve at a rapid pace, due in large part to advancements in computational technology that have enabled new avenues for investigating clinical challenges. Deep learning (DL) and statistical shape modelling (SSM) are two such technologies with the potential to create new ways of addressing clinical questions in orthopaedic research. Specifically, computational pipelines that harness the two in a synergistic fashion have particular promise. This review describes how DL and SSM can be effectively employed to investigate orthopaedic clinical research questions and enhance patient care and surgical outcomes. While the applications of each of these technologies are nearly infinite, this review will focus primarily on how these technologies enable large‐scale analysis of bony morphology to gather valuable insights into the prediction of clinical outcomes, using examples from the surgical management of anterior cruciate ligament (ACL) reconstruction and shoulder instability. We begin by discussing each technology individually, then expand on their integration towards enhancing the capabilities of one another, in a synergistic manner.

## DL IN ORTHOPAEDIC RESEARCH

DL is a subset of artificial intelligence (AI) that utilizes neural networks with multiple layers that can learn and make predictions from large amounts of unstructured data, such as images [4, 5]. In orthopaedic research, DL has shown emerging potential in automating the analysis of medical imaging data, including X‐rays, computed tomography (CT) scans and magnetic resonance imagings (MRIs) [3].

Given the growing role of peri‐operative imaging, the development of DL has been of great interest in terms of assisting the clinical integration of advanced imaging analyses. For instance, after ACL reconstruction, DL algorithms can be trained to analyze MRI images to detect subtle changes in the bone and surrounding tissues that might not be apparent to the naked eye [1]. For instance, DL models have been explored to predict the likelihood of complications, such as graft failure or post‐traumatic osteoarthritis development, eventually enabling tailored patient‐specific rehabilitation protocols and improving long‐term outcomes. In the case of shoulder instability, DL may assist in outcome evaluation by analyzing pre‐ and post‐operative imaging to assess as a substrate for understanding risk factors that may indicate the need for soft tissue or bone augmentation in terms of predicting the success of surgical stabilization, or the positioning of surgical implants. Taken together, the above examples serve to emphasize that access to automated image analyses reduces the large manual burden associated with these advanced analyses, which in turn fosters a more efficient workflow for clinical integration and objectifies the assessment of advanced imaging as part of the orthopaedic work‐up. This efficiency not only contributes to accelerating research but also works to enhance clinical decision‐making.

One recent application of DL in this area involved the development of a DL‐based pipeline that enables automatic segmentation of the scapula on MRI, generating a three‐dimensional (3D) model that is comparable to those generated from CT [8]. Such a technology has the potential to allow surgeons to obtain all clinically relevant information from MRI as a standalone study in the work‐up of shoulder instability—rather than the current need for a separate CT scan to evaluate bony morphology—inherently reducing the need for multiple imaging studies for patients with shoulder pathology. This, in turn, decreases the cost of clinical work‐ups and limits radiation exposure.

## SSM IN ORTHOPAEDIC RESEARCH

Although less well‐known than DL, SSM is another powerful technology that can be applied independently of or in complement to DL in orthopaedic research [9]. SSM involves the construction of mathematical models that represent variations in the shape of anatomical structures within a population [9]. By analyzing a large number of bone shapes, SSM can identify patterns and correlations that contribute to understanding the factors influencing surgical outcomes, providing valuable insight into optimizing patient care. Importantly, SSM does not rely on a priori‐defined assumptions and can thus be leveraged as a computational opportunity for revealing new insights into the assessment of bony morphology that go beyond long‐held assumptions in orthopaedics.

In the case of ACL injury, SSM can be used to study the anatomical variations in the knee joint across different patients [1, 6, 9]. These models can identify how specific shape features of the femur and tibia influence the risk of ACL injuries and their subsequent surgical outcomes. For example, Polamalu et al. applied SSM to assess the 3D bony morphology of distal femurs and proximal tibiae of ACL‐injured knees, the contralateral uninjured knees of ACL‐injured subjects, and knees with no history of injury [6]. The authors created surface models by segmenting bone from bilateral CT scans of 20 subjects with ACL‐injured knees and non‐injured contralateral knees and 20 knees of control subjects with no history of a knee injury. Principal component analysis—a technique that simplifies complex data sets by transforming them into a set of new variables, called principal components, that capture the most important patterns and reduce the dimensionality of the data while preserving as much variation as possible—was used to determine modes of anatomical variation [6]. They found that ACL‐injured knees had a more lateral femoral mechanical axis and a greater angle between the long axis and condylar axis of the femur and that a smaller anterior–posterior dimension of the lateral tibial plateau was associated with ACL‐injured knees [6]. Such an approach to understanding bony morphology emphasized the clinical utility of advanced imaging analyses in the context of understanding bony morphology and its underlying relationship to predict clinically relevant factors such as predisposition to ACL injuries.

While relatively less work has investigated the use of SSM for shoulder instability, one can imagine the ways by which SSM can help in assessing the glenoid cavity's shape and its impact on surgery outcomes, for example, references [2, 7]. By creating a statistical model of the glenoid cavity from a large patient cohort, for example, researchers can pinpoint which shape characteristics are associated with successful stabilization and which ones correlate with recurrent dislocations. This knowledge could help guide the design of patient‐specific implants and surgical procedures, ultimately improving stability and function.

**Figure 1:**
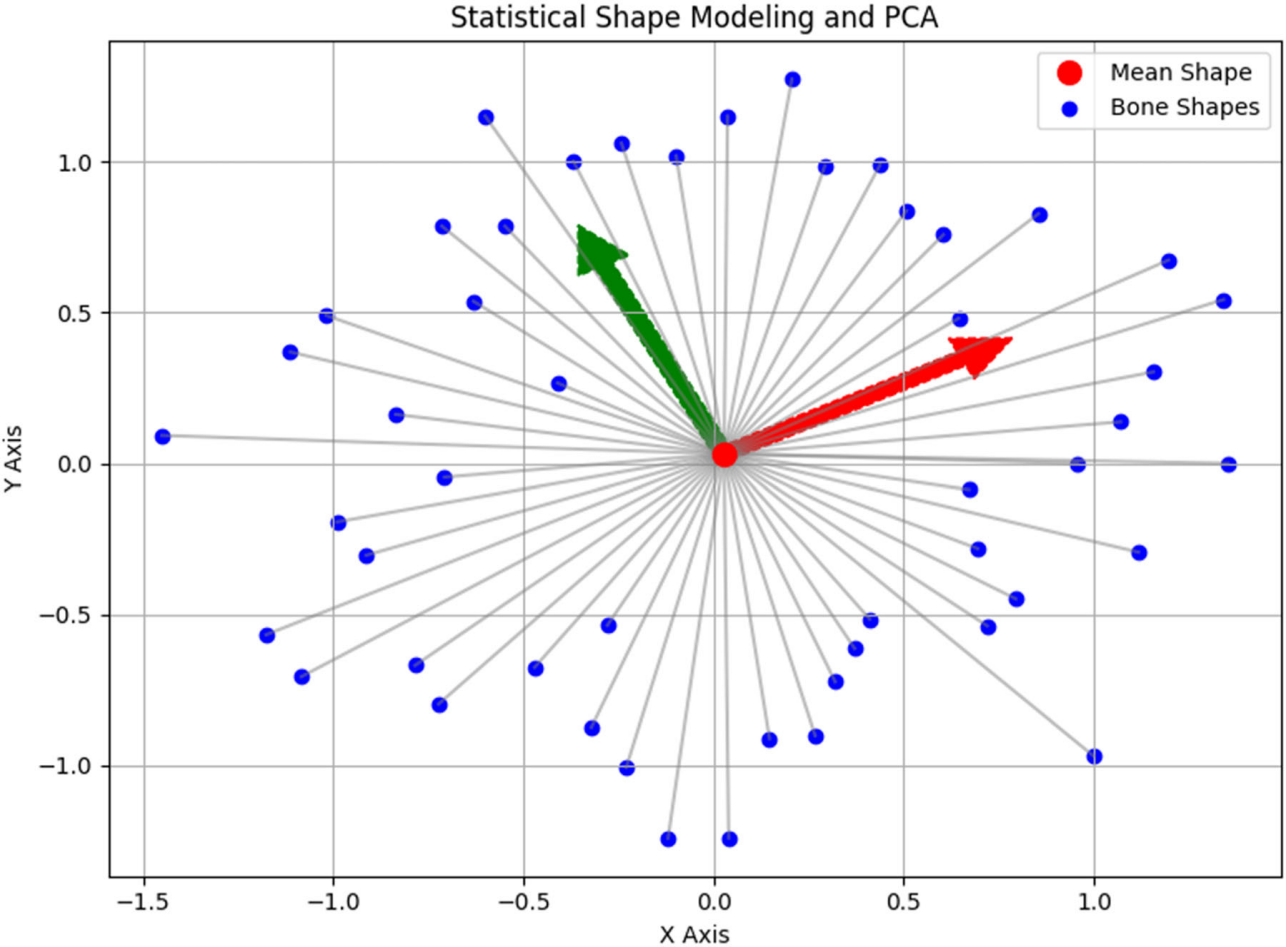
Simplified demonstration of statistical shape modelling (SSM) and principal component analysis (PCA). The scatter plot represents simplified, synthetic variations in bone shapes (blue points), each representing a distinct anatomical variation. The red dot denotes the mean shape, calculated from the dataset, serving as a central reference point. Grey lines connect individual bone shapes to the mean shape, highlighting deviations and demonstrating how each shape contributes to the overall variability. PCA identifies the principal components (PC1 and PC2) that best explain the variance in the dataset. In the context of SSM and bone shape analysis, principal components refer to the main axes or directions of variability within a dataset of bone shapes. PCA identifies these principal components by finding the eigenvectors of the covariance matrix of the data. Each principal component is associated with an eigenvalue, which represents the amount of variance explained by that component. Arrows in red (PC1) and green (PC2) indicate the directions of maximum shape variation, providing insights into dominant patterns of bone morphology. Such an approach facilitates the quantification and visualization of complex anatomical variations and is a powerful tool for understanding bone shape features associated with pathology.

Figure 1 provides a simplified demonstration of how SSM and principal component analysis may be applied to understand bone shape variations associated with pathology.

## INTEGRATING DL AND SSM

The integration of DL and SSM offers a synergistic approach to orthopaedic research, particularly when it comes to our understanding of bony morphology and its relationship to perioperative planning with the objective of optimizing post‐operative outcomes. DL provides a means to automate the extraction of shape features from medical images (through automated segmentation of imaging data, for example), providing high‐dimensional data that can feed into SSM pipelines. Likewise, SSM can enhance DL models by providing a comprehensive understanding of shape variability and its clinical implications. Taken together, these technologies facilitate a more robust and detailed analysis of bone shape features on a large scale, in the context of their clinical relevance for direct application. For example, in the context of investigating ACL reconstruction outcomes, DL algorithms can be trained to segment and classify different regions of the knee joint from a very large number of MRIs (i.e., femur, tibia, patella, cartilage, ACL and menisci). The segmented images can then be analyzed using SSM to understand how specific shape variations in these structures correlate with post‐operative outcomes. Such a combined approach would allow for the identification of predictive biomarkers and the development of personalized surgical plans. While such a pipeline could also be developed without DL, it is important to note the key role of DL in such a pipeline, as it enables the automated segmentation and extraction of a much larger number of MRIs than would be feasibly or efficiently possible through manual segmentation alone. Similarly, in shoulder instability surgery, one could imagine how the integration of DL and SSM could enable the creation of personalized 3D models of the shoulder joint, which could be used to simulate different surgical scenarios and predict outcomes. Such predictive modelling could substantially enhance preoperative planning, leading to more precise and effective surgical interventions over time.

## CONCLUSION

The application of DL and SSM to orthopaedics has substantial potential to enhance research around joint bony morphology and its effect on patient outcomes. Advancements in these fields have created opportunities for large‐scale, detailed analyses of bone shape features and may provide critical insights that can improve surgical outcomes and patient care. As these methods continue to evolve, their integration will likely become widespread across orthopaedic research and drive forward the development of more effective, personalized treatments.

## AUTHOR CONTRIBUTIONS

*Study design, data acquisition, data analysis, data interpretation, manuscript drafting and critical revision*: All authors.

## CONFLICT OF INTEREST STATEMENT

The authors declare no conflict of interest.

## ETHICS STATEMENT

Not applicable.
